# Cryptic iron cycling influenced by organic carbon availability in a seasonally stratified lake

**DOI:** 10.1093/femsec/fiaf029

**Published:** 2025-03-20

**Authors:** Verena Nikeleit, Markus Maisch, Daniel Straub, Sümeyya Eroglu, Jimena C Lopez-Rivoldi, Harald Strauss, Fin Ring-Hrubesh, James M Byrne, Andreas Kappler, Casey Bryce

**Affiliations:** Department of Geoscience, University of Tübingen, 72076 Tübingen, Germany; now: NORCE Norwegian Research Center AS, 5008 Bergen, Norway; Department of Geoscience, University of Tübingen, 72076 Tübingen, Germany; Quantitative Biology Center (QBiC), University of Tübingen, 72076 Tübingen, Germany; Institute for Geology and Paleontology, University of Münster, 48149 Münster, Germany; Department of Geoscience, University of Tübingen, 72076 Tübingen, Germany; Institute for Geology and Paleontology, University of Münster, 48149 Münster, Germany; School of Earth Sciences, University of Bristol, Bristol, BS8 1RJ, UK; School of Earth Sciences, University of Bristol, Bristol, BS8 1RJ, UK; Department of Geoscience, University of Tübingen, 72076 Tübingen, Germany; Cluster of Excellence: EXC 2124: Controlling Microbes to Fight Infections, 72076 Tübingen, Germany; School of Earth Sciences, University of Bristol, Bristol, BS8 1RJ, UK

**Keywords:** phototrophic Fe(II) oxidation, photoferrotrophs, Fe(III) reduction, dimictic lake

## Abstract

Iron cycling including phototrophic Fe(II) oxidation has been observed in multiple permanently stratified meromictic lakes, yet less focus has been on dimictic lakes, which seasonally overturn and are vastly more common. Here, we investigated iron cycling in a dimictic lake, Großes Heiliges Meer in northwest Germany, using 16S rRNA amplicon sequencing, as well as *in-situ* and lab-based experiments. Bacterial community composition in the lake follows geochemical gradients and differs markedly between oxic and anoxic conditions. Potential iron-metabolizing bacteria were found mostly in anoxic conditions at 7 and 8 m depth and were comprised of taxa from the genera *Chlorobium, Thiodictyon, Sideroxydans, Geobacter*, and *Rhodoferrax*. We were able to recreate active iron cycling (1) with an *ex-situ* microbial community from 8 m depth and (2) with a successful microbial enrichment culture from 7 m depth. Varying the light and organic carbon availability in lab-based experiments showed that Fe(III) reduction overshadows Fe(II) oxidation leading to a cryptic iron cycle. Overall, we could demonstrate that microbial iron cycling can be a key biogeochemical process in dimictic lakes despite regular disturbance, and that complex environmental factors such as organic substrates control the balance between Fe(II) oxidation and Fe(III) reduction.

## Teaser

High availability of carbon in seasonally stratified lakes hides the contribution of phototrophic bacteria in driving the oxidation of iron.

## Introduction

Iron cycling, consisting of Fe(II) oxidation and Fe(III) reduction, is a widespread process in stratified lakes and freshwater sediments, and plays a critical role in shaping the biogeochemistry of lacustrine environments (Berg et al. 2016, Otte et al. 2018, Kappler et al. 2021). During Fe cycling and subsequent formation and dissolution of Fe minerals, nutrients (e.g. phosphorus), carbon, and pollutants can be bound and sequestered or released back into the environment (Tipping 1981, Eickhoff et al. 2014, Mu et al. 2016, Kappler et al. 2021). Furthermore, various microbial metabolisms link iron redox transformations with those of other key element cycles including carbon, sulfur, and nitrogen (Kappler et al. 2021). Both oxidative and reductive parts of the iron cycle can be facilitated abiotically and biotically. However, microbial iron cycling plays a particularly important role under microoxic and anoxic conditions where rapid abiotic oxidation by O_2_ is inhibited (Bryce et al. 2018, Kappler et al. 2021).

Fe(II) oxidation in oxic environments is either abiotically driven by reaction with atmospheric O_2_ or is microbially mediated at low pH (Kappler et al. 2021). In the interface of oxic to anoxic environments, Fe(II) can be directly oxidized by microaerophilic Fe(II)-oxidizing bacteria (Kucera and Wolfe 1957, Maisch et al. 2019). In contrast, in anoxic, (sunlit) environments, nitrate-reducing and phototrophic bacteria like *Chlorobium* spp., *Thiodictyon* spp., and *Acidovorax* spp. can oxidize Fe(II) (Heising et al. 1999, Croal et al. 2004, Kappler et al. 2005, Laufer et al. 2017, Bryce et al. 2018). Direct microbial Fe(III) reduction occurs in anoxic environments and is performed by various genera of bacteria i.e. *Geobacter* spp., *Shewanella* spp., and *Geothrix* spp. (Myers and Nealson 1990, Lovley et al. 1993, Coates et al. 1999), whereas indirect microbial Fe(III) reduction occurs in sulfur-rich environments where bacteria reduce oxidized sulfur-species to form sulfides which react abiotically with Fe(III) to form FeS or/and FeS_2_ (Canfield 1989).

Both Fe(III)-reducers and Fe(II)-oxidizers have been found to be abundant and active in freshwater sediments and stratified lakes (Berg et al. 2016, Laufer et al. 2016, Otte et al. 2018). However, much of the previous work on anaerobic iron cycling in freshwater environments has focused on permanently stratified meromictic lakes due to their relevance as an analogue to Archean ocean biogeochemistry (reviewed in Bryce et al. 2018, Kappler et al. 2021). Meromictic lakes do not mix completely and typically develop an anoxic bottom water body (monimolimion) that is not mixed and thus creates stable geochemical gradients (Stewart et al. 2009). For example, an active iron cycle with potential for rapid FeS recycling has been found in meromictic Lake Cadagno (Switzerland) which has low iron content (Berg et al. 2016); as well as in Lake Matano (Indonesia) and Lake La Cruz (Spain) which contain hundreds of micromolar dissolved Fe(II) in the anoxic bottom waters (Crowe et al. 2008, Walter et al. 2014). A number of these lakes contain active phototrophic Fe(II)-oxidizers (Crowe et al. 2008, Walter et al. 2014). True meromictic lakes are relatively rare as their existence requires specific physical and geochemical conditions e.g. the basin is particularly deep and steep-sided; or there are particularly steep salinity differences between layers that inhibit full water-column overturn even when temperatures are favourable. In contrast, most lakes are holomictic (uniform from top to bottom) where the salinity is constant throughout the depth. Variations in thermal stratification drive water density fluctuations, causing holomictic lakes to mix regularly. These may be classed as polymictic, where the lake mixes multiple times a year, monomictic (mixes once) or dimictic, which mixes twice per year usually in spring and autumn. These seasonally stratified lakes have been gaining increasing attention as hot-spots for microbial iron cycling due to their relative abundance and apparent suitability for supporting a diversity of iron-based metabolisms (Schiff et al. 2017, Tsuji et al. 2020, Liu et al. 2022).

Carbon also plays a key role in Fe(II) oxidation and Fe(III)-reduction. Phototrophic Fe(II)-oxidizers can use inorganic carbon (CO_2_) and different organic carbon molecules like acetate, lactate and others as a carbon source (Ehrenreich and Widdel 1994, Heising et al. 1999, Jiao et al. 2005). Additionally, they can use organic molecules such as acetate as an electron donor (McKinlay and Harwood 2010). During their growth, phototrophic Fe(II)-oxidizers can use multiple electron donors like Fe(II) and organics simultaneously or in sequential order depending on the different electron donors and different Fe(II)-oxidizers (Ehrenreich and Widdel 1994, Melton et al. 2014, Nikeleit et al. 2024). The same organic molecules are also used by Fe(III)-reducers and are necessary for their growth (Lovley et al. 1993, Pinchuk et al. 2009). Thus, organic carbon plays an important role in both sides of iron cycling. Recent research of the impacts of organics on iron cycling and minerals has been reviewed by Dong et al (2023), and a study by Peng et al (2019) that demonstrated influences of organic carbon on iron cycling with a phototrophic Fe(II)-oxidizer and abiotic Fe(III) reduction. Yet the impact carbon has on microbial iron cycling in complex environments such as lakes is largely unknown.

In this study, we investigated iron cycling in a dynamic lake and assessed the role of carbon in dictating the relative contribution of microbial Fe(II) oxidation and Fe(III) reduction in shaping biogeochemical cycling. Specifically, we present insights on Fe cycling from Großes Heiliges Meer in northwest Germany, a dimictic lake that develops anoxic bottom layers containing iron and sulfur during the summer months (mid-April to October). It is surrounded by valley sand containing Fe-bearing minerals like garnet and epidote underlain by gypsum-containing Münder-Mergel formation (Dölling and Stritzke 2009). Using a suite of *in-situ* and *ex-situ* experiments we investigated the relative importance of Fe(II)-oxidizing and Fe(III)-reducing processes throughout the depth profile at summer stratification; and assessed how changes in carbon input alter microbial iron cycling.

## Materials and methods

### Field site

Großes Heiliges Meer is a dimictic lake in northwest Germany near Hopsten at 52°21′06.73′′N and 7°38′03.22′′E. It is part of a well-established field station in a protected area of the Landschaftsverband Westfalen-Lippe Museum für Naturkunde Münster (Museum of Natural History Münster). The lake has a maximum depth of 10.8 m and an average depth of 4.4 m. It is always stratified in winter and between mid-April to late October (Pott 2009, Swanner et al. 2022). Sampling took place September 9th 2021.

### Geochemical data

Temperature and oxygen content were measured with an *in-situ* electrode (ThermoScientific Orion Star) at the desired depths on September 9th 2021. Water samples for dissolved Fe, sulfate (SO_4_^2−^) and dissolved inorganic carbon (DIC) analyses were pumped from the desired depths and prepared on board for later measurements. Water samples were filtered (0.45 µm) into pre-cleaned 50-ml tubes and 5 drops of 65% HNO_3_^−^ added to keep Fe dissolved. Total dissolved Fe and Mn were measured in the lab with ICP-OES (SpectroFlame-EOP, SPECTRO Analytical Instruments). Another 12 ml of filtered water samples were measured with ion chromatography (761 compact IC, Firma Methrom AG) to determine SO_4_^2−^ concentrations and DIC was determined by titration of 0.1 M HCl with 100 ml filtered water sample to pH 4.3. DOC was analysed by combustion at 750°C (Elemental analyser, multi N/C 2100S, Analytik Jena GmbH, Germany).

### Community analysis

To assess changes in microbial community composition over the depth of the lake 16S rRNA amplicon sequencing was conducted at 4 different depths. Selected water depths were at 3, 5, 7, and 8 m. On September 9th, 2021, water was pumped up from the desired depth and collected in a sterile 5 L canister, filled to the top to avoid air bubbles. Eight samples (duplicate per depth) were prepared for DNA extraction, 16S rRNA (gene) amplicon sequencing and analysis as described below as soon as possible and always on the same day as collection.

### DNA extraction

Samples were filtered through SterivexTM-GP sterile filter (0.22 µm, Merck KGaA, Darmstadt, Germany) until the filter was blocked. Two different filters were used for each depth (for community analysis) and for each setup of the *in-situ* experiment at 7 m depth so that duplicate samples for sequencing were obtained. The filters were stored in sterile bags at −20°C until DNA extraction was performed. Afterwards, the filter paper was removed from the filter and DNA was extracted using the UltraClean R Microbial DNA Isolation Kit (MO BIO Laboratories, Carlsbad, CS, USA). The quantity of the DNA was measured with a Nanodrop ND-1000 Spectrometer (Nanodrop™ 1000, Thermo Scientific, Waltham, MA, USA).

### 16S rRNA gene amplicon sequencing and analysis

DNA was amplified using forward primer 16S-515F and reverse primer 16S-806R (Caporaso et al. 2011) targeting the V4 region of the 16S ribosomal RNA gene. Library preparation steps (Nextera, Illumina) and 250 bp paired-end sequencing with MiSeq (Illumina, San Diego, CA, USA) using v2 chemistry were performed by Microsynth AG (Balgach, Switzerland). Between 9925 and 118 334 read pairs were obtained for each of the samples (in total 5 124 544 read pairs). One replicate of an *in-situ* sample originating from 8 m—“8 m a” had the lowest number of reads (9925), all other samples had more than 39 000. Sequencing data were analyzed with nf-core/ampliseq v2.3.1, which encompasses all necessary analysis steps and software. The pipeline is publicly available (Ewels et al. 2020, Straub et al. 2020), and was executed with Nextflow v21.10.3 (Di Tommaso et al. 2017) and singularity v3.8.7 (Kurtzer et al. 2017). Primers were trimmed, and untrimmed sequences were discarded (<36% per sample) with Cutadapt version 3.4 (Martin 2011). Adapter and primer-free sequences were processed with DADA2 v1.22.0 (Callahan et al. 2016) to eliminate PhiX contamination, trim reads (before median quality drops below 35; forward reads were trimmed at 230 bp and reverse reads at 207 bp), correct errors, merge read pairs, and remove polymerase chain reaction chimeras; ultimately, 5218 amplicon sequencing variants (ASVs) were obtained across all samples. Taxonomic classification was performed with DADA2 and the SILVA v138 database (Quast et al. 2012). Intermediate results were imported into QIIME2 version 2021.8.0 (Bolyen et al. 2019). Four hundred fifty-eight ASVs classified as chloroplasts or mitochondria were removed, totaling 0% to 50.2% (average 12.7%) relative abundance per sample, and retaining 4760 ASVs across all samples. The final abundance table had 4375 to 81 690 read counts per sample (total 3 110 373 read counts). The lowest read counts had one replicate of an *in-situ* sample originating from 8 m—“8 m a”, all other samples had >19 000. Alpha rarefaction curves were produced with the QIIME2 diversity alpha-rarefaction plugin, which indicated that the richness of the samples had been fully observed. Raw sequencing data has been deposited at NCBI in the Sequence Read Archive under BioProject accession number PRJNA1157215 (https://www.ncbi.nlm.nih.gov/bioproject/PRJNA1157215).

### *In-situ* community responses to Fe(II) and organic input at 7 m depth

To investigate potential for *in-situ* Fe(II) oxidation, we conducted an incubation experiment in the lake itself. Water was pumped from a depth of 7 m into a sterile plastic cannister until the bottle was full and closed to avoid oxygen exposure on September 9th 2021. Water was then filled into 1 l acid-cleaned and sterile Schott bottles and the headspace (100 ml) was degassed with N_2_ for 5-10 min. Four different experimental setups were conducted in duplicates each. The experimental conditions tested were: (1) no addition of substrates (as a control), (2) with the addition of 2 mM Fe(II), (3) the addition of Fe(II) and an organic carbon mix (0.6 mM of acetate and lactate each), and (4) with the addition of the organic mix only. All four conditions were conducted both with and without 10 µM of DCMU (3-(3,4-dichlorophenyl)-1,1-dimethylurea; C_9_H_10_Cl_2_N_2_O) added to inhibit cyanobacteria and thereby abiotic oxidation of Fe(II). The bottles were then placed back into the lake at 7 m depth, to be removed at each sacrificial sampling point. In total, three sampling time points were chosen. For time zero, water from 7 m depth was collected. For time point 2 and 3, the prepared 1 l bottles were placed back into the lake at depth 7 m for 3 (time point 1) and 23 days (time point 2). At each time point, the water in the bottles was analysed for Fe(II)/Fe(III) ratios, acetate and lactate concentrations, and DNA was extracted for microbial community analysis.

### Iron cycling experiment with the *ex-situ* community from 8 m depth

Water for incubation experiments was collected from water depth of 8 m on September 9th 2021. Water was pumped into a sterile plastic cannister and filled to the top to avoid air bubbles and, thus, oxygen exposure. In the laboratory, 50 ml of the water was filled into sterile 100 ml serum vials and prepared for three different setups. Setup 1 was amended with 2 mM Fe(II); setup 2 with 2 mM Fe(II) and 1 mM organic mix (0.5 mM acetate and 0.5 mM lactate); and setup 3 with 2 mM organic mix (1 mM acetate and 1 mM lactate). For every setup, three biological bottles were prepared and one abiotic control (addition of 15 mM formaldehyde). Bottles were placed for the first part of the experiment into an incubator with 14 h light and 10 h dark at 8°C to mimic field conditions. After 33 days, conditions were changed to 24 h dark to favour Fe(III) reduction and suppress phototrophic Fe(II) oxidation and after 44 days changed back to 24 h illumination to promote phototrophic Fe(II) oxidation. Samples were collected in a glovebox with 100% N_2_ atmosphere for Fe analysis and quantification.

### Fe(II) oxidation and Fe(III) reduction rates of enrichment culture from 7 m depth

An enrichment culture was later obtained from the *in-situ* experiment from 7 m depth in the setup where only Fe(II) was added. The enrichment was grown over several transfers in constant light with Fe(II) and bicarbonate buffer with low phosphate media at 20°C and N_2_/CO_2_ (90%/10%) (Ehrenreich and Widdel 1994). The enrichment was subsequently maintained in continuous culture for multiple transfers under these conditions. Three experimental conditions were tested in triplicates with all bottles randomly placed in a 20°C light incubator. The experimental setups contained either (1) 2 mM Fe(II) to study Fe(II) oxidation rates, (2) 2 mM synthesized ferrihydrite (Fe-Red-Fh) with 1 mM of lactate and acetate each, or (3) biogenic Fe(III) oxyhydroxides (2 mM) (Fe-Red-Bios) with 1 mM of lactate and acetate to study Fe(III) reduction rates. Biogenic Fe(III) oxyhydroxides for setup 2 were obtained from pre-incubated bottles with the enrichment first doing Fe(II) oxidation and after full Fe(II) oxidation were prepared to enhance Fe(III) reduction by adding organics and wrapping the bottle in aluminium. Biotic Fe-oxyhydroxides were used to simulate the Fe-oxides found in the environment. Bottles for setup 1 and 2 were wrapped in aluminium foil. Samples for Fe and high performance liquid chromatography (HPLC) were taken anoxically and stored at 5°C for quantification.

### Iron cycling experiment with enrichment culture from 7 m depth

The enrichment culture was transferred to low phosphate media with 2 mM Fe(II) and placed randomly in a light incubator at 20°C for six transfers and sequencing was done after four transfers (Ehrenreich and Widdel 1994). Once all Fe(II) was oxidized the bottles were wrapped in aluminium foil to inhibit phototrophs and 2 mM lactate was added. After all Fe(III) was reduced, the bottles were unwrapped again and light illuminated. Samples for Fe and HPLC were taken anoxically and stored at 5°C for quantification.

### Fe quantification

Samples measuring 0.1 ml for Fe quantification were taken anoxically and directly aliquoted into 0.9 ml 1 M HCl. Samples were stored at 4°C until measurements and Fe(II) and total Fe were quantified with the Ferrozine assay after Hegler (Hegler et al. 2008).

### Fatty acid analysis

After sampling, samples were centrifuged at 15 000 rpm for 10 min to remove cells and minerals, and the supernatant was transferred to a new Eppendorf tube and stored at 4°C until analysis. HPLC analysis was performed with a Shimadzu Prominence HPLC with a LC-20ATsolvent delivery unit, CTO-10ASvp column oven and a RID-20a refractive index detector.

### Phylogenetic tree

The 16S rRNA gene sequences of *Thiodictyon* from Großes Heiliges Meer were compared to reference *Thiodictyon* 16S rRNA gene sequences and model Fe(II)-oxidizer strains downloaded from GenBank. Alignment of the isolate and reference gene sequences was performed using ClustalW (Thompson et al. 1994). A phylogenetic tree was then constructed in MEGA11 using the Maximum-Likelihood method based on the Tamura-Nei model and 1000 bootstrap replicates were used to validate the topological structure (Tamura et al. 2021).

## Results

### Geochemical profile of Großes Heiliges Meer

**Figure 1.**
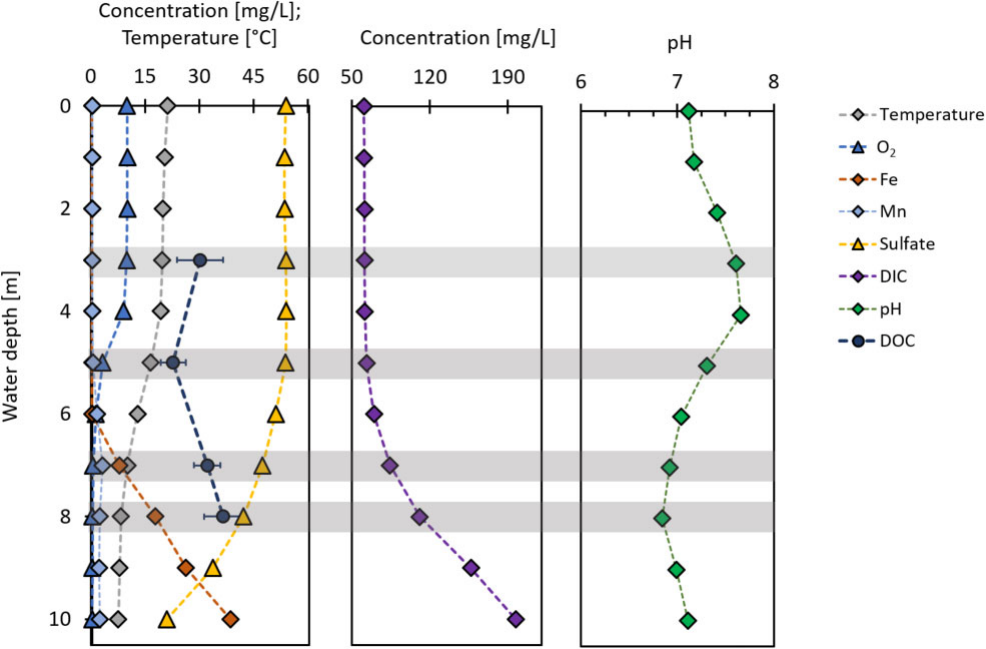
Geochemical data of temperature, O_2_, Fe, Mn, SO_4_^2−^, DIC, DOC, and pH from the water column of Heiliges Meer. Grey background indicates depth where samples for community analysis were taken.

The temperature at the surface was 20.9°C and decreased continuously to 7.2°C at 10 m depth. pH was 7.1 at the surface and increased at 3-4 m depth to 7.6 before decreasing down to 7.0 again at 6 m depth (Fig. 1). Oxygen content started to decrease at 3 m depth from 9.68 mg/l to under 0.3 mg/l at 7 m depth. At 7 m, 7.5 mg/l (0.13 mM) dissolved total Fe was detected for the first time and increased to 38 mg/l (0.7 mM) Fe at 10 m depth. Dissolved Mn could be quantified at 6 m (1.4 mg/l) and increased to 2.0 mg/l at 10 m depth. In the same depth as, dissolved Mn started to increase (5 m), SO_4_^2−^ concentration decreased by 40% at 10 m compared to 6 m, from 53 mg/l at 6 m to 20.6 mg/l at 10 m depth. Additionally, an increase in DIC was detected from 63 mg/l at 5 m to 200 mg/l at 10 m depth. DOC values range from 30 ± 6 mg/l at 3 m to 32 ± 4 mg/l at 7 m and 36 ± 5 mg/l at 8 m.

### Microbial community composition in Großes Heiliges Meer

**Figure 2.**
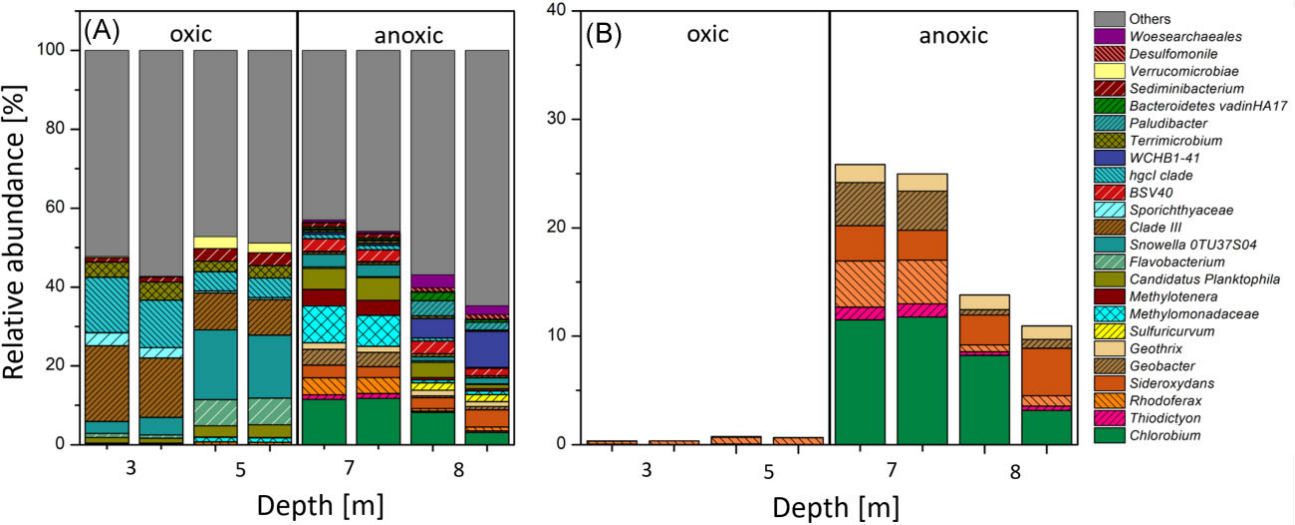
*In-situ* 16S rRNA sequencing of four depths from Großes Heiliges Meer. Panel A shows bacterial community for 3, 5, 7, and 8 m water depth in duplicates. Taxa are shown individually if relative abundance was 1% or higher. Panel B shows abundances of potential iron metabolizing genera (family for Clade III, BSV 40, Methylomonadaceae, Sporichthyaceae, Bacteroidetes vadinHA17 and order for WCHB1-41, Verrucomicrobiae, if no genera were available for 3, 5, 7, and 8 m water depth).

DNA was extracted from 3, 5, 7, and 8 m depth and analysed in duplicate. Differences in the microbial community were observed between 3/5 and 7/8 m depth (Fig. 2). In 3 m and 5 m depth, aerobic and facultatively anaerobic microorganisms (*hgcI clade, Flavobacterium*), and heterotrophs (*Clade III*) were found alongside cyanobacteria (*Snowella*). Once oxygen is depleted, the bacterial community shifted at 7 and 8 m depth to be dominated by strict anaerobes like sulfur-reducers (*Desulfomonile*), sulfur-oxidizers (*Sulfuricurvum*), methane-oxidizers (*Methylomonadaceae, Methylotenera*), fermenters (*Terrimicrobium*), and potential Fe(II)-oxidizers and Fe(III)-reducers. The potentially Fe(II)-oxidizing community members are species of *Chlorobium* and *Thiodictyon* (phototrophic Fe(II)-oxidizers), and *Sideroxydans* (microaerophilic Fe(II)-oxidizer). Potential Fe(III)-reducers identified included representatives of *Rhodoferax, Geobacter* and *Geothrix*. A clear difference in relative abundance was observed for potential iron-metabolizing bacteria between oxic and anoxic conditions in the lake. At 3 and 5 m depth (oxic conditions), iron-metabolizing bacteria were 0.4 to 0.8% of the bacterial community, whereas at 7 and 8 m depth (anoxic conditions) they comprised 25.5 and 12% of the total bacterial community. In general, in anoxic conditions at Großes Heiliges Meer, bacteria associated with iron, sulfur, methane, and carbon metabolism have been found in the anoxic waters and show a diverse community linked to many environmentally relevant cycles.

### *In-situ* community responses to Fe(II) and organic input at 7 m depth

**Figure 3.**
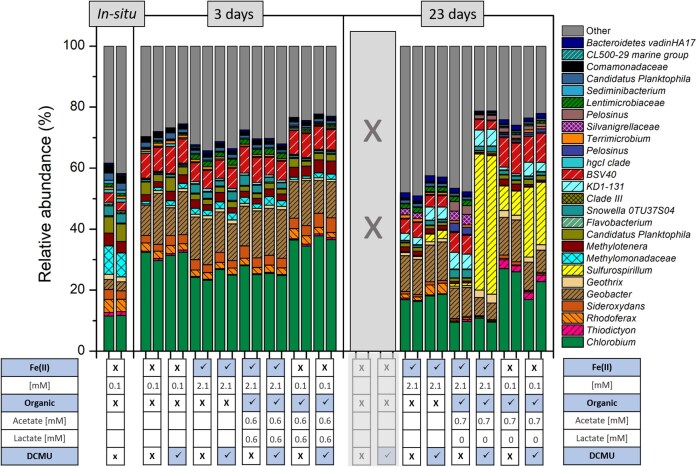
Bacteria community of in-situ experiment from initial community (*in-situ*), 3 and 23 days are shown in duplicates. Bacteria genera are shown individually if relative abundance was 1% or higher. (family for Clade III, BSV 40, Methylomonadaceae, Sporichthyaceae, Bacteroidetes vadinHA17, and order for WCHB1-41, Verrucomicrobia). Table underneath indicates different substrate addition (Fe(II), organic or/and DCMU).

We conducted *in-situ* experiments to (1) establish whether the observed Fe(II)-oxidizing microorganisms are active in the lake and (2) to test whether the presence of DOC inhibits Fe(II) oxidation *in-situ* given the observed phototrophic Fe(II)-oxidizers are known to be metabolically flexible (Heising et al. 1999, Melton et al. 2014, Bryce et al. 2018, Nikeleit et al. 2024). One litre Schott bottles with water from 7 m depth were spiked with relevant substrates and placed back at 7 m depth for 3 and 23 days. After 3 days, no change in Fe(II)/Fe(tot) was measured, as well as no change in acetate and lactate concentrations (Fig. 3). The bacterial community was also similar to the initial time point. Generally, an increase of *Chlorobium* (from 11% to 31%) and *Geobacter* (from 3% to 12%) and a decrease in *Thiodictyon, Rhodoferax, Geothrix*, and methane-oxidizers (from 12% to 3%) was observed. Highest abundance of potential Fe-metabolizing bacteria was found in the control, organic and organic+DCMU setups. For the samples collected at 23 days, no control data are presented as the control bottles were, unfortunately, trapped on the buoy and could not be retrieved from the lake. No change in Fe(II)/Fe(tot) could be observed in all setups. Lactate, where present, was completely used up and acetate increased to 0.7 mM. Generally, a decrease of *Chlorobium* (from 31% to 18%), methane oxidizers (from 3% to 0.7–1.8%) and *Siderooxidans* (<1%) were observed compared to day 3. In the setups with DCMU and/or organics, an increase of *Sulfurospirillum* up to 45% (in Fe(II)+organic+DCMU) was observed. In the setup with Fe(II)+organic+DCMU, organic and organic+DCMU an increase in *Geothrix* (up to 3%) was also observed. Additionally, in the setups amended with acetate/lactate an increase of *Thiodictyon* (up to 3%) was observed.

### Iron cycling experiment with *ex-situ* microbial community from 8 m depth

**Figure 4.**
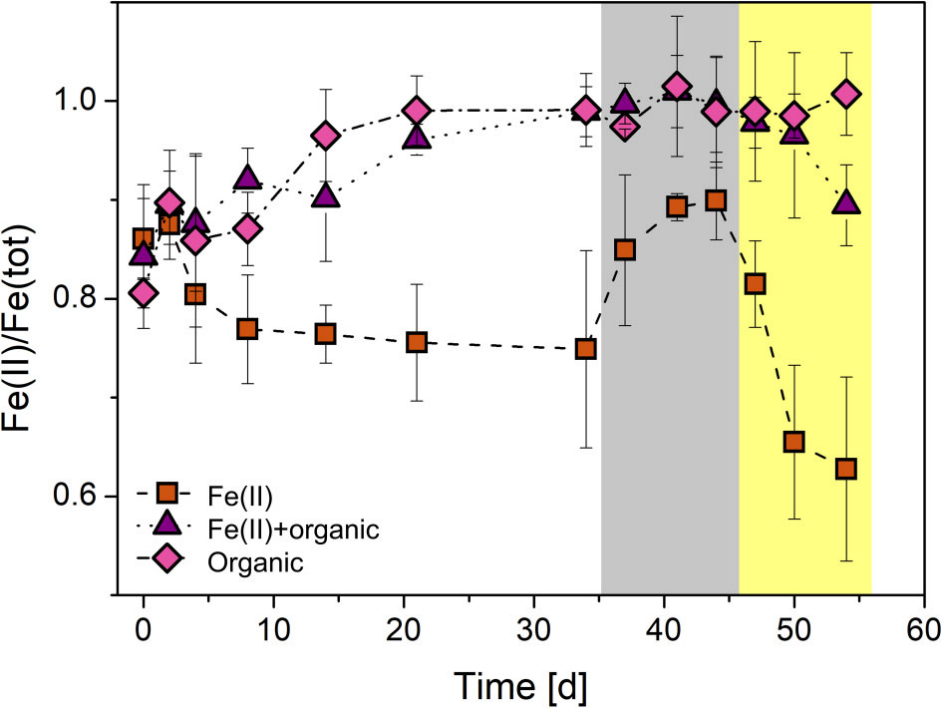
Iron cycling experiment from 8 m depth of Heiliges Meer with different setups. Brown square shows Fe(II) only setup, purple triangle shows Fe(II) plus organic setup and pink diamond shows organic only setup. For the first 34 days all setups were exposed to a 14/10 h light/dark cycle. Afterwards the bottles were placed for 10 days in the dark (grey background) and then 10 days into the light (yellow background). Standard variations were calculated from triplicates.

Both the microbial community analysis and the geochemical profile of the water column suggest that phototrophic Fe(II) oxidation and Fe(III) reduction could be taken place in parallel at the depth of the chemocline at 8 m. To further investigate the effect of the day-night exposure experienced by the *in-situ* microbial community, as well as to investigate the effect of organics we setup experiments under laboratory conditions. We set up three different treatments where water from 8 m was amended with only Fe(II), Fe(II)+organic or organic and incubated under anoxic conditions in triplicates. All three setups started with Fe(II)/Fe(tot) ratios of around 0.83 and were exposed to a 14/10 h light/dark cycle at 8°C to mimic *in-situ* conditions at the time of sampling. Both the Fe(II)+organics and organic setup showed Fe(III) reduction and reached a Fe(II)/Fe(tot) ratio of 0.99±0.03 and 0.99±0.04 respectively after 34 days (Fig. 4). In the setup amended with Fe(II), slight Fe(II) oxidation occurred and reached a Fe(II)/Fe(tot) ratio of 0.75±0.1 after 34 days. To further study the individual parts of the iron cycle, namely to promote only Fe(III) reduction or Fe(II) oxidation, the setups were placed in darkness for 10 days and afterwards for 10 days in the light. In the Fe(II)+organics and organic setup, no change in Fe(II)/Fe(tot) was measured during this period, whereas Fe(III) reduction was observed in the Fe(II) only setup and Fe(II)/Fe(tot) increased to 0.90±0.04. After 44 days all three setups were placed in a light incubator with constant illumination and no change in Fe(II)/Fe(tot) was observed in the organic setup. In the Fe(II)+organic and Fe(II) setup, Fe(II) oxidation could be observed in both cases and reached 0.90±0.04 (Fe(II)+organic) and 0.63±0.09 (Fe(II) only) Fe(II)/Fe(tot) after 54 days.

### Fe cycling with an enrichment culture from 7 m

**Figure 5.**
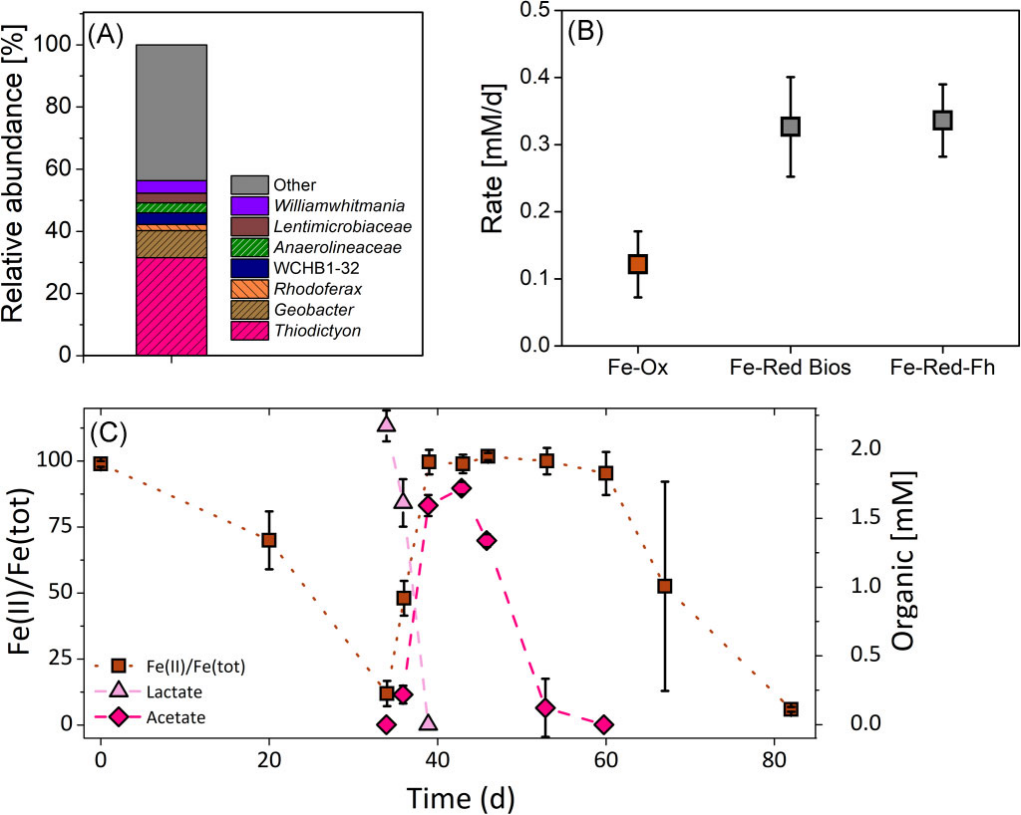
16S rRNA sequencing and iron cycling of phototrophic Fe(II)-oxidizing enrichment from Großes Heiliges Meer. Panel A shows bacterial community of a phototrophic enrichment culture originated from 7 m depth of Großes Heiliges Meer (genera shown, family for Lentimicrobiaceae and Anaerolineaceae). Panel B shows Fe(II) oxidation and Fe(III) reduction rates. Fe-Red Bios indicate Fe(III) reduction rate with biotic Fe(III)oxyhydroxides and Fe-Red-Fh indicate Fe(III) reduction rates with synthesized ferrihydrite. Rates were calculated from triplicates. Panel C shows cycling experiment with phototrophic enrichment whereas brown rectangle shows Fe(II)/Fe(tot), light pink triangle lactate concentration and dark pink diamond acetate concentration. Standard deviation was calculated from triplicates.

We successfully maintained an enrichment culture from the *in-situ* experiment at 7 m in the setup with Fe(II) only addition. The enrichment grew under autotrophic conditions with 2 mM Fe(II) as electron donor and continuous light illumination. 16S rRNA gene amplicon sequencing showed that one single *Thiodictyon* ASV was the dominant bacteria (32%) (Fig. 5A). This potential Fe(II)-oxidizer was also found in the *in-situ* community from 7 m. The culture also contained potential Fe(III)-reducers; (1) a sequence from the genus *Rhodoferrax* which represents 2% and (2) two different sequences annotated as *Geobacter* that, combined, represent 9% of the community (Fig. 4). Other bacteria were strictly chemotrophic anaerobes from the genera *Anaerolineaceae, Lentimicrobiaceae*, and *Williamwhitmania*. Given the abundance of potential Fe(II)-oxidizers and Fe(III)-reducers we set up experiments promoting phototrophic Fe(II) oxidation and Fe(III) reduction. In the setup promoting phototrophic Fe(II) oxidation with 2 mM Fe(II) and no organics present, a lag phase of 9 days was observed and the experiment ended after 19 days with 90% of Fe(II) oxidized (Fig. 5B). It should be noted that one triplicate bottle had a longer lag phase and its Fe(II) oxidation rate was lower than the other two. The average Fe(II) oxidation rate reached 0.12±0.05 mM/d. Experiments to promote Fe(III) reduction were set up with two different initial Fe(III) minerals: (1) with biogenic Fe(III) oxyhydroxides and (2) with synthetic ferrihydrite. Fe(III) reduction rates in both setups were similar reaching 0.33±0.07 (biogenic Fe(III) oxyhydroxides) and 0.34±0.05 mM/d (synthetic ferrihydrite) and the colour changed from orange to black in both cases (Fig. 5B). Compared to the Fe(II) oxidation rates, Fe(III) reduction rates were almost three times higher. To simulate iron cycling the enrichment was placed first into constant light and all Fe(II) was oxidized after 34 days, then the enrichment was wrapped in aluminium foil and 2 mM lactate were added (Fig. 5C). While Fe(III) was reduced completely at day 39, lactate was completely consumed and acetate was produced up to 1.7 mM. The enrichment was placed into constant light again but no Fe(II) was oxidized until day 60. Instead consumption of acetate was observed and once consumed at 60 days, Fe(II) oxidation continued and was completely oxidized after 82 days. However, large variations in Fe(II) oxidation rates between the triplicates were observed.

## Discussion

### Key phototrophic Fe(II)-oxidizer in Großes Heiliges Meer

**Figure 6.**
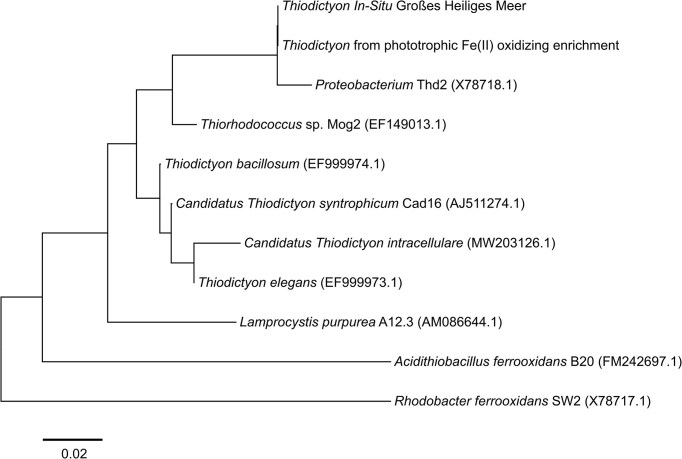
A maximum-likelihood tree based on 16S rRNA gene sequences indicating the phylogenetic position of Thiodictyon from this study among reference Thiodictyon sequences and model Fe(II)-oxidizers. Scale bar for branch length indicates the number of substitutions per site. Accession numbers are included in brackets.

In Großes Heiliges Meer, the only taxa attributed to potential phototrophic Fe(II) oxidation were *Chlorobium*, a green sulfur bacteria (12% at 7 m), and *Thiodictyon*, a purple sulfur bacteria (1% at 7 m). Few isolated *Chlorobium* from stratified lakes have the ability to oxidize Fe(II). From Lake la Cruz, a *Chlorobium*-dominated enrichment culture was obtained (Walter et al. 2014), from Lake Kivu the first pelagic strain *C. phaeoferrooxidans* KB01 isolated (Crowe et al. 2017) and a co-culture dominated by *Ca.C. masyuteum* was enriched from Brownie lake in Minnesota (Lambrecht et al. 2021). Although the *in-situ* community of potential photoferrotrophs was dominated by a *Chlorobium* with 12% in Großes Heiliges Meer, the phototrophic enrichment was obtained with a *Thiodictyon* (1% *in-situ* community). This is the first time that an enrichment capable of Fe(II) oxidation was successful for a dimictic lake with a dominant Fe(II)-oxidizer being a *Thiodictyon* (31% of enrichment) and not a *Chlorobium. Thiodictyon* belongs to the *Chromatiaceae* family of the *Gammaproteobacteria* class. In a phylogenic tree we tested how close the *Thiodictyon* in our study is to other *Thiodictyon* and other Fe(II)-oxidizing model strains like *Acidithiobacillus ferrooxidans* B20 and *Rhodobacter ferrooxidans* SW2. We could determine that the *Thiodictyon* ASV from the lake is the same *Thiodictyon* ASV that was enriched in the phototrophic Fe(II) oxidation enrichment (Fig. 6). The *Thiodictyon* in our study is closely related (98.43%) to another *Thiodictyon* Thd2 associated to phototrophic Fe(II) oxidation (Ehrenreich and Widdel 1994). *Lamprocystis purpurea* A12.3, *Candidatus Thiodictyon syntrophicum* Cad16 and *Thiorhodococcus* sp. Mog2 are other purple sulfur bacteria found in stratified lakes (Lunina et al. 2005, Peduzzi et al. 2012). *Lamprocystis purpurea* A12.3 and *Candidatus Thiodictyon syntrophicum* Cad16 were found in Lake Cadagno, where Berg et al (2016) studied FeS recycling. FeS recycling was tested in a lab incubation of an *in-situ* community from Lake Cadagno where FeS was added as the sole electron donor and after 6 months the enrichment was dominated by bacteria morphologically similar to *Thiodictyon* (Berg et al. 2016). *Thiodictyon* has also been associated with acetate assimilation (Kappler and Newman 2004). Acetate could potentially be assimilated by *Thiodictyon* in the setups with organics in the *in-situ* experiment (Fig. 3). After day 23 an increase of *Thiodictyon* could be observed in comparison to the *in-situ* community in the setup where only organics were added. In the experiment with the phototrophic Fe(II)-oxidizing enrichment, we could demonstrate that phototrophic Fe(II) oxidation was performed by a phototrophic enrichment dominated by *Thiodictyon* (Fig. 5). *Thiodictyon* is the only bacteria in the enrichment associated to both phototrophy and Fe(II) oxidation. Thus, we suggest *Thiodictyon* has strong potential to conduct phototrophic Fe(II) oxidation in Großes Heiliges Meer. Nevertheless we cannot rule out the participation of *Chlorobium* in iron cycling or its association with other cycles like the sulfur cycle (Thompson et al. 2017).

### Potential for a cryptic iron cycle and effect of organics

Despite the clear potential for microbial Fe(II) oxidation, particularly the abundance of anoxygenic phototrophs, it is surprising that no changes in Fe speciation were observed during the *in-situ* experiment. Furthermore, all measured iron was in the reduced Fe(II) form. This could have two plausible explanations. Either (1) there are no active bacteria present that can oxidize iron or (2) all oxidized iron is immediately reduced soon after it is oxidized resulting in no net change in Fe speciation. We favour the latter interpretation, which would also align with the calculated rates of Fe(III) reduction and Fe(II) oxidation from our enrichments where Fe(III) reduction rates were ∼3 times higher than Fe(II) oxidation rates (0.33 and 0.12 mM/d, respectively).

Additionally, we found both potential Fe(II)-oxidizers and Fe(III)-reducers in Großes Heiliges Meer at 7 and 8 m depth (Fig. 2) demonstrating clear potential for a complete iron cycle. The sequencing data suggest that anoxygenic photoferrotrophs from the genera *Chlorobium* and *Thiodictyon* could play a crucial part in Fe(II) oxidation below the metalimnion. However, our enrichment culture only contained *Thiodictyon*, thus whether or not the *Chlorobium* contribute to Fe(II) oxidation is unclear. Microaerophilic Fe(II)-oxidizers like *Sideroxydans* are also present at 7 and 8 m and could contribute to Fe(II) oxidation. We did not sample the community during the night, but it is likely that microaerophilic Fe(II)-oxidizers would play a relatively more important role in Fe(II) oxidation in the dark given that photoferrotrophy would be inhibited during the night (Nikeleit et al. 2024). Above the metalimnion, Fe(II) is likely oxidized abiotically by O_2_ produced by the cyanobacteria present (*Snowella*, Fig. 2).

On the Fe(III) reduction side four different potential Fe(III)-reducers were found. *Geobacter* can couple the oxidation of acetate to Fe(III) reduction (Lovley et al. 1993, Caccavo et al. 1994), whereas *Rhodoferax* and *Geothrix* can couple Fe(III) reduction to the oxidation of lactate and acetate (Coates et al. 1999, Finneran et al. 2003, Risso et al. 2009). Another potential Fe(III)-reducer could be *Sulfurospirillum* which couples Fe(III) reduction to oxidation of sulfur and thiosulfate (Straub and Schink 2004). From the geochemical data alone we cannot draw conclusions as to which Fe(III)-reducer and which Fe(II)-oxidizers actively contribute to Fe turnover and to which extent. In the enrichment culture, however, we measured lactate consumption during Fe(III) reduction (Fig. 6) and identified *Rhodoferax*. The coupled consumption of lactate with the production of acetate has been observed in Fe(III)-reducer and could be an indication for microbial Fe(III) reduction (Pinchuk et al. 2009). Fe(III) reduction could also be indirectly taken place by sulfate-reducing bacteria and abiotic Fe(III) reduction of sulfide to FeS minerals (black minerals observed in enrichment experiment).

This potential for a cryptic iron cycle was further demonstrated in our iron cycling experiments with the *ex-situ* community from 8 m depth. We observed that Fe(II) oxidation and Fe(III) reduction are stable in a day/night cycle with no added organics (Fig. 4, Fe(II)-only setup). When organics were added Fe(III) reduction was stimulated and became the dominating process and thereby masking Fe(II) oxidation (Fig. 4). It could also be that *Thiodictyon*, like other phototrophic Fe(II)-oxidizer, is able to use organics and could use both organics and Fe(II) at the same time or in sequential order (Nikeleit et al. 2024). Once the organics are used up Fe(II) oxidation becomes the dominating process (Fig. 4, Fe(II)+organic and Fe(II) only setup and Fig. 5). It is hard to distinguish and exclude processes and it could be that all are taking place at the same time. The results in our study demonstrate that photoferrotrophs are abundant and active at Großes Heiliges Meer, yet their contribution is masked by a faster Fe(III)-reducing community which can thrive on the organics present and rapidly recycle Fe(III).

### Limitations of studying *in-situ* microbial iron cycling

In our experimental design, we intended to eliminate the potential contribution of cyanobacteria to Fe(II) oxidation in the light by adding the inhibitor DCMU. In all of these setups, bacteria from the genus *Sulfurospirillum* bloomed, reaching up to 45% of the bacterial community. It is possible that DCMU provided an additional source of carbon and nitrogen which could be accessed by these bacteria. This drastic shift was not observed when we added Fe(II), acetate and lactate, suggesting that these substrates play an active role and did not trigger a fundamental change in the microbial community composition. In future, alternative methods should be used to assess the impact of microbial iron oxidation in the absence of cyanobacteria.

### Dimictic lakes as potential iron cycling habitats

So far, studies on phototrophic Fe(II) oxidation, iron cycling and Archean ocean analogues have focused on permanently stratified meromictic lakes such as Lake Kivu (Llirós et al. 2015), Brownie Lake (Lambrecht et al. 2021), Lake Cadagno (Berg et al. 2016), Lake de la Cruz (Walter et al. 2014), Lake Pavin (Busigny et al. 2014) and Lake Matano (Crowe et al. 2008, 2017). Of all lakes on earth, only a few are characterized as meromictic whilst most lakes are holomictic and mix periodically. Dimictic lakes like Großes Heiliges Meer, mix once in spring and once in fall. During the summer stratification anoxic bottom water can be formed. Geochemical gradients of oxygen depletion and iron rich bottom waters have been observed in Großes Heiliges Meer, as they are observed in some other dimictic lakes such as Lake 227 and 442 (Kenora, Canada). These conditions make them suitable refugia for phototrophic Fe(II)-oxidizers which once dominated the Earth's oceans and are now resigned to more isolated anoxic yet sunlit niches (Liu et al. 2022, Schiff et al. 2017, Swanner et al. 2022). Geochemical data from Großes Heiliges Meer from 2014, 2015, and 2018 are comparable to the observations in this study with oxygen depletion at 6 to 7 m (Swanner et al. 2022). 16S rRNA amplicon sequencing data of Großes Heiliges Meer showed that potential Fe(II)-oxidizing and Fe(III)-reducing bacteria represented up to 25% of the microbial community in the anoxic bottom waters. This implies that despite regular turnover a diverse iron-cycling microbial community can rapidly re-form when geochemical conditions are favourable. The combination of *in-situ* experiments, and iron cycling experiments with the *ex-situ* community and the enrichment culture could confirm that iron cycling takes place in Großes Heiliges Meer; both Fe(III) reduction and phototrophic Fe(II) oxidation. This study shows that complete iron cycling can take place in dimictic lakes but is likely dominated by Fe(III)-reduction, creating a cryptic cycle in which Fe(II) oxidation can be easily overlooked.

## Conclusions

In this study, we could show that dimictic lakes like Großes Heiliges Meer are habitats for microbial iron cycling, despite the regular disruption of their geochemistry during seasonal turnover. This further expands the refugia in which photoferrotrophs in particular can survive, demonstrating they can form active communities in holomictic lakes as well as in the rarer meromictic lakes. However, we also demonstrated that the contribution of microorganisms to Fe(II) oxidation is likely masked by a cryptic iron cycle driven by fast and efficient Fe(III)-reducing bacteria. The availability and quality of DOC is thus a critical control on the balance of Fe(II) oxidation and Fe(III) reduction in dimictic lakes; controlling whether Fe(III) (oxyhydr)oxides are stable and thus able to interact with carbon, nutrients and pollutants. Alongside enhancing our fundamental understanding of such important iron cycling habitats, we were able to enrich an iron-metabolizing culture dominated by the phototrophic Fe(II)-oxidizer *Thiodictyon*. This is the first time that iron cycling could be simulated in a dimictic lake using the *ex-situ* community and an enrichment culture.

## Supplementary Material

fiaf029_Supplemental_File
